# Spiral readouts for 4D flow MRI

**DOI:** 10.1186/1532-429X-14-S1-W31

**Published:** 2012-02-01

**Authors:** Andreas Sigfridsson, Sven Petersson, Carl Johan Carlhall, Tino Ebbers

**Affiliations:** 1 Division of Cardiovascular Medicine, Department of Health Sciences, Linköping University, Linköping, Sweden; 2 Center for Medical Image Science and Visualization (CMIV), Linköping University, Linköping, Sweden; 3 Clinical Physiology, Linköping University Hospital, Linköping, Sweden

## Summary

The feasibility of using spiral readouts to reduce the scan time of 4D flow MRI without sacrificing data quality was investigated.

## Background

Time-resolved three-dimensional three-directional phase contrast MRI (4D flow) is being increasingly used to study the physiology and patophysiology of cardiovascular blood flow. However, the key limitation of existing implementations is the long scan times required. Spiral readouts are efficient and have appealing flow sensitivity properties. In this work, we investigate whether the scan time can be reduced by using spiral readout trajectories without reducing data quality.

## Methods

A stack-of-spiral 4D flow pulse sequence was implemented on a 1.5T MRI scanner with identical spatiotemporal resolution as a conventional Cartesian pulse sequence (2.8mm isotropic voxel size, 48.8ms temporal resolution). The thoracic aorta of ten healthy volunteers were imaged using both pulse sequences. The Cartesian acquisition used SENSE factor 2, whereas the spiral acquisition did not use parallel imaging. The spiral acquisition used water-selective excitation, TE/TR 3.5/12ms with 12 spiral interleaves. Navigator gating with a window of 5mm was used to suppress respiratory motion, and VENC was set to 1.5m/s for both techniques. The spiral acquisition was evaluated by comparing through-plane volume flow with the Cartesian technique. The ascending aorta was reformatted and segmented manually. Additionaly, the flow data quality was assessed by analyzing the number of pathlines that crossed the geometric boundaries of the aorta. Pathlines were emitted backwards from descending aorta in order to avoid sensitivity to branching flow. Any errors in the flow data, including background phase errors, eddy currents, noise and blurring, will accumulate over the trajectory integration and trajectories that does not conform to the geometry thus indicate errors in the data.

## Results

**Figure 1:**
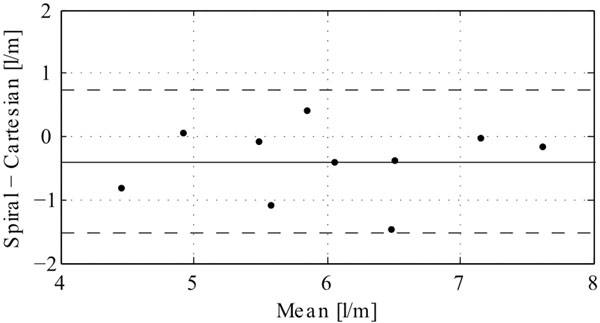
Bland-Altman analysis of the cardiac output measured using the spiral and the conventional Cartesian technique. The bias (solid line) was -0.40, and the limits of agreement (dashed lines) were ±1.13 l/min.

**Figure 2:**
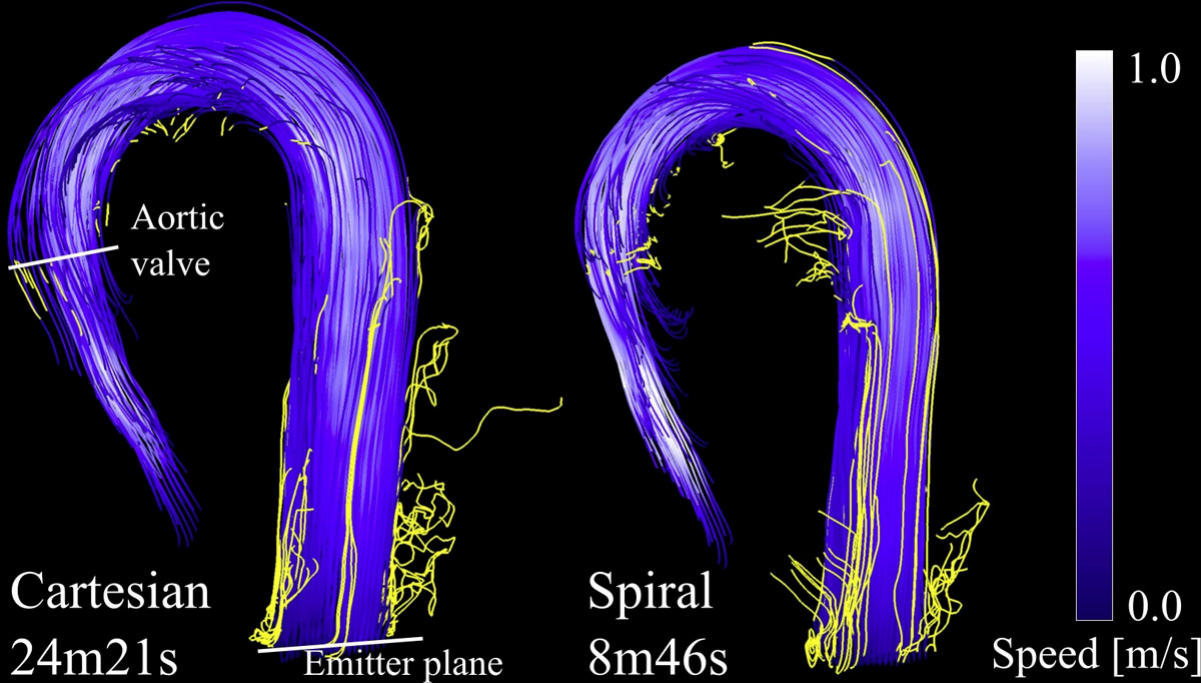
Pathlines generated from the data using both the conventional Cartesian technique and the spiral technique. The pathlines were traced backwards starting from the descending aorta, in order to avoid sensitivity to branching flow. Wall clock scan times (including the navigator efficiency) are indicated for this volunteer. Pathlines that leave the geometrical boundaries of the aorta (yellow) are detected and used as a measure for data fidelity to compare the two methods.

The scan times were 14m57s±4m36s for the Cartesian acquisition and 6m12s±1m16s for the spiral acquisition, including navigator gating. There was no difference between the cardiac output measured using the Cartesian acquisition compared to the spiral acquisition (6.2±1.0 vs 5.8±1.0 l/min, NS). A Bland-Altman plot of the cardiac output is shown in Figure 1. The bias was -0.40, and the limits of agreement were ±1.13 [l/min]. The number of traces that were confined within the aortic boundaries during the pathline trace duration of 326±40ms was 51±16% for the Cartesian acquisition and 60±7% for the spiral acquisition (NS). The pathlines of one of the volunteers are shown in Figure 2.

## Conclusions

Using spiral readouts, the scan times could be reduced more than two-fold without sacrificing data quality. The spiral acquisition used in this work did not yet include parallel imaging, which could be used to further reduce the scan time. The shorter scan time may open up for broader use of 4D flow MRI for the assessment of cardiovascular flow in health and disease.

## Funding

The Swedish Research Council, and the Swedish Heart-Lung Foundation.

